# Mr. Perry's *Case of Delirium Tremens*

**Published:** 1814-01

**Authors:** Charles James Perry

**Affiliations:** Member of the Royal College of Surgeons in London. Bristol


*To the Editor of the Medical and Physical Journal.*

SIR,

A STRONGLY marked case of that disease denominated by Dr. Sutton Delirium Tremens, having lately come under my observation, I feel much satisfaction in the task of reporting it to the medical world, through the medium of your Journal.

On the 21st of last month a physician and myself were sent for to Mr. P. a wine cooper, of an athletic figure and fair complexion, aged 34 years. We found him in a sitting room, dressed in black. He arose with a hurried movement and violent shaking of the arms and legs. His countenance was rather expressive of anxiety for something lost, than of wild fierceness; his eye, although suffused, was neither red nor sparkling; his hearing not impaired; his face rather pale than florid, and frequently covered with large drops of sweat; and the jaw a little dropt. He spoke, as he moved, in a manner both hurried and confused. His whole appearance and manner conveyed to me the idea of a man about to suffer for a great crime, in the moment he was in expectation of a reprieve. He had been raving when we were sent for, and endeavoring to escape from the house, under the impression that he was going to attend a funeral; and had dressed himself in black for the occasion. He fancied his pockets full of fowls, and busied himself in picking imaginary insects from his cloaths, the appearance of which he described as

 similar similar to small mice. He mistook his nearest relations for other people, and his friends had been obliged to use force to keep him quiet; indeed, so unmanageable had he been before we saw him, as to require three or four men to hold him, and it had become absolutely necessary to secure the doors and windows, to prevent his escaping into the street; but to the doctor and myself he was tolerably rational, and perfectly condescending.

The tremors of the arm, and the starting of the tendons at the wrist, made it difficult to count the pulse, which was very unequal, or appeared so, both as to strength and fullness; but it exceeded 150 strokes in the minute. He would not acknowledge any pain in the head, although his friends said he had complained of it. He observed to us that he had pains in the limbs, and pointed to the precordia. He had not slept at all during four days and nights, and complained much of the want of sleep. His bowels were open; urine scanty and red; tongue white, and rather furred; considerable thirst; and the face and neck unusually moist. His habits for some years had bordered on daily intoxication, and he was much addicted to smoking tobacco. Some weeks ago he had been briskly purged on account of a contusion of the arm, by the medical gentleman who then attended him. Under all these circumstances, it was determined to draw sixteen ounces of blood from the arm, which was readily accomplished, after the assistants had sufficiently steadied it, which proved a difficult task, as the tremors, or rather spasmodic twitching, were so violent and incessant. The opening was made, and the blood flowed with unusual force and rapidity: as it passed he became more tranquil, and the sweating from the face and neck ceased; but in a few minutes after the arm was bandaged, the trembling and hurried manner returned. The physician with whom I had the satisfaction to attend, observed that this disease bore much similarity to that lately described by Dr. Sutton under the name Delirium Tremens; and considered it a fair opportunity for putting his practice to the test, in which opinion I perfectly coincided. One grain of pure opium was immediately given, and repeated every second hour: in the evening the patient had taken five of the one-grain pills, but was rather more hurried, more confused, and more delirious; with a pulse, if possible, more rapid and unequal. He was now ordered a grain of opium every hour. On the morning of the next day he had taken seven more pills, in all twelve grains, without any material amendment. Four draughts were now ordered him, each containing four grains of opium, one of which he was to take every third hour; and as there remained three one-grain opium pills, he was directed to take them also, if he did not fall asleep before night. He took the four draughts and the pills, which, with the former, made *thirty-one grains* of opium in less than *thirty-six hours.* About nine at night he fell asleep, from which in some hours he awoke perfectly sensible, and without tremors. Pulse 104, soft, full, and steady; tongue clean; urine natural; bowels open; and the sweat gone. A draught containing three grains of opium was given at bed-time. On the following day the tremors slightly returned, with some aberration of mind. Three draughts were prescribed him, each containing four grains of opium, all of which he took before midnight. The tremors still continuing perceptible, an infusion of valerian root, with camphor, was ordered: this he took without any apparent good effect. Four grains of opium were taken at bed-time, and the like dose repeated at midnight, which procured him some little sleep. He still remained much indisposed, and the opium was this day (the 26th) altogether omitted. On the 27th, at bed-time, he took five grains of crude opium in the form of pill, and the like quantity was repeated the following night. About twelve o'clock he fell asleep, and continued sleeping, with little interruption, until five the ensuing evening, in all seventeen hours, when he awoke, perfectly free from tremor—calm—and collected. We discontinued our visits from the 4th instant, having the satisfaction to leave him perfectly restored in mind and body. I had some conversation with him yesterday (the 11th), and find he has not had the least return of his complaint.

It is remarkable that through the disease the appetite was good; and that after the first grand exhibition of the opium, the pulse became nearly natural, the tongue clean, the skin free, without partial sweats, the urine of the usual color, and the bowels continued soluble. During the first eight days he took *sixty-four grains of opium.*

In the sketch given of this case, I think there is sufficient shown to establish a diagnosis from phrenitis; a point of the highest importance; for, as the practical distinction is great, the diagnosis should be clear, and to prevent mistake the names of the two diseases should bear no resemblance to each other. I therefore would prefer the name adopted by Dr. Sutton, although it may be objectionable, to that lately proposed in a criticism on Dr. Sutton's work.

I have the honor to be, Sir,

Your obedient Servant,

CHARLES JAMES PERRY,

*Member of the Royal College of Surgeons in London.*

